# The use of immunotherapy treatment in malignant pheochromocytomas/paragangliomas: a case report

**DOI:** 10.1186/s13256-021-02733-5

**Published:** 2021-03-29

**Authors:** Robin Raquel Rodriguez, Saleha Rizwan, Khaled Alhamad, Gene Grant Finley

**Affiliations:** Department of Medical Oncology, Allegheny General Hospital, Pittsburgh, PA 15212 USA

**Keywords:** Pheochromocytomas, Immunotherapy, Oncology, Rare tumors, Off-label treatment options, Case report

## Abstract

**Background:**

Pheochromocytomas are a subset of paragangliomas, which are a rare group of neural crest cell-derived tumors. Malignant cases of both pheochromocytomas and paragangliomas are even rarer, and currently there is no standard of care. This case report details the use of off-label immunotherapy and its efficacy in the management of the aforementioned tumor.

**Case presentation:**

Herein is presented a case of a 60-year-old Caucasian female with a rare malignant pheochromocytoma. The tumor was determined to be unresectable because of involvement of surrounding organs. Radiation therapy was also not a viable option because of concerns over appreciable toxicity in relation to mass size. As there is no standard of care for malignant cases, the patient was started on chemotherapeutic agents but was soon shown to be intolerant to this treatment. As she was ineligible for several clinical trials, the patient was started on the off-label immunotherapeutic agents nivolumab and ipilimumab. Immunotherapy use resulted in decreased tumor size, improved quality of life, and reconsideration for radiation therapy.

**Conclusions:**

The use of immunotherapy in pheochromocytoma in this patient clearly demonstrated substantial benefit, as she was able to be reconsidered for radiation therapy. Not only has the patient been tolerant of this treatment, but she has exhibited progression-free survival of over 20 months. As there is no current standard treatment for malignant pheochromocytomas, the success of its use in this patient lends support to the ongoing clinical trials regarding the use of immunotherapy in rare tumors, including pheochromocytomas.

## Introduction

It is estimated that two to eight people out of 1 million will be diagnosed yearly with either pheochromocytomas or paragangliomas [1]. Out of these cases, malignant pheochromocytomas (PHEOs) are even rarer and make up around 10% of all pheochromocytoma cases, with up to 25% of paragangliomas (PGLs) being considered malignant [2, 3].

As these tumors are catecholamine-producing neuroendocrine tumors, they usually present as incidental findings related to hypertension [4]. They often arise between the ages of 30 and 50 years and can be associated with multiple endocrine neoplasia (MEN2B), von Hippel–Landau (VHL) disease, neurofibromatosis (NF), and renal cell carcinoma (RCC), among others [1]. It is estimated that approximately 40% of all PHEOs/PGLs involve a germline mutation [1]. Treatment of PHEOs/PGLs often involves resection of the tumor for local cases, although there is still no current standard of care for malignant/metastatic cases. Currently, for unresectable/metastatic cases, radiation therapy such as external-beam radiation therapy (ERBT), nonsurgical ablative therapy, and chemotherapy are the treatment options of choice [3]. For symptomatic patients with functional tumors, octreotide/lanreotide is often used for management of symptoms [3]. Other treatment options used in the management of unresectable/metastatic cases include lutetium Lu 177 dotatate (Lutathera) and iobenguane I-131 along with tyrosine kinase inhibitors such as sunitinib and pazopanib [5]. Immunotherapy (IO), which acts on various pathways in the immune system, has been used for a wide variety of metastatic cancers. Recent studies and ongoing clinical trials are being completed to evaluate the efficacy of IO for PHEOs/PGLs [6, 7]. Nevertheless, at present, it still remains only an off-label treatment option [8].

## Case

**Fig. 1:**
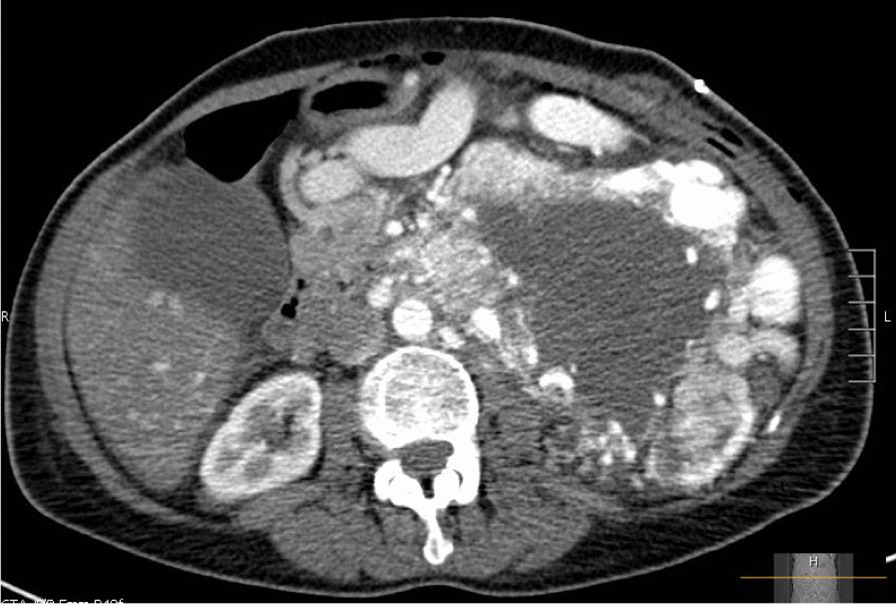
CT scan demonstrating left renal mass

This case is of a 60-year-old Caucasian female with no significant past medical history who initially presented to her primary care physician for pharyngitis symptoms. During the visit an incidental murmur was discovered, leading to an echocardiogram that revealed thrombus formation in the right atria and inferior vena cava. Subsequent computed tomography (CT) scan of the chest, abdomen, and pelvis depicted an 18.7 × 14.1 cm left renal mass with extension into the inferior vena cava (IVC) and renal and splenic vein with encompassment of the small bowel and pancreas (Fig. 1). The patient continued to deny any symptoms apart from sore throat and weight loss. She was started on anticoagulation. Two weeks after initial presentation, a radical open nephrectomy was attempted, but the mass was unresectable as a result of extensive tumor fixation onto surrounding organs. A biopsy demonstrated a highly cellular neoplasm that was diffusely positive for synaptophysin, chromogranin, and GATA-3 transcription factor on immunohistochemistry. Histology revealed a highly cellular neoplasm with moderate nuclear pleomorphism and hyperchromasia. Additionally, two to three mitotic figures were noted per ten high-power fields, noting the potential for malignant behavior of the tumor. Pathology findings and multidisciplinary discussion led to suspicion of pheochromocytoma over renal cell carcinoma. A metaiodobenzylguanidine (MIBG) scan was conducted and confirmed the diagnosis of pheochromocytoma. Subsequent positron emission tomography (PET) and brain magnetic resonance imaging (MRI) ruled out metastatic disease. Because of the patient’s lack of typical endocrinological PHEO symptoms (such as hypertension, diaphoresis, and dyspnea) and normal urine metanephrine levels, the tumor was considered to be nonfunctional. Genetic testing was also performed, with no variant mutations found in the rearranged during transfection (*RET*), VHL succinate dehydrogenase complex subunit C (*SDHC*), succinate dehydrogenase complex iron sulfur subunit B (*SDHB*), or succinate dehydrogenase complex subunit D (*SDHD*) genes. She was clinically staged as stage III (cT3, cNX, cM0).

As a result of the thrombus, initial hospitalization course was complicated by congestive heart failure, and atrial fibrillation stabilized with rate control management. Heparin-induced thrombocytopenia also developed, requiring change of anticoagulation to a novel anticoagulant (NOAC). The patient was discharged 3 weeks following aborted nephrectomy. Initiation of systemic therapy was discussed but was determined to not be the best option for the patient at the time. Aortography and angiography were performed, demonstrating extensive tumor vascularity with feeding vasculature from the celiac, superior mesenteric, inferior mesenteric, and lumbar arteries. Thus, treatment with interventional radiology (IR) embolization was performed with the intent of diminishing tumor vascularity and reducing thrombus formation. IR embolization was performed twice, 2 months apart. It was discontinued when CT scans revealed no significant change of the tumor mass or thrombus. The patient was also evaluated by the radiation oncology department, who determined that she was ineligible for radiation therapy. This was due to concerns over the size of the mass and the appreciable toxic effects that could arise from radiating such a large area.

**Fig. 2:**
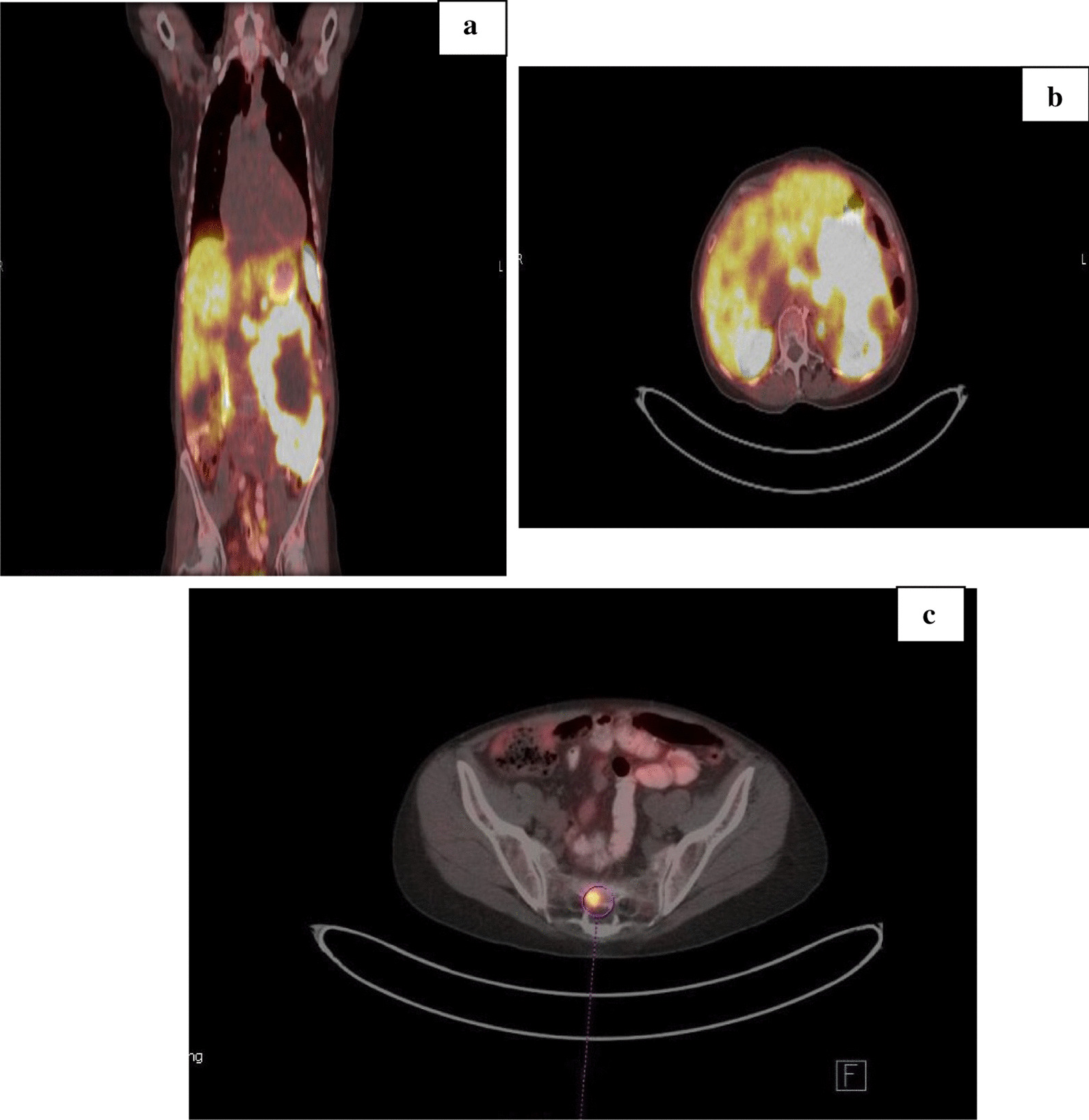
Positron emission tomography (PET) scan (**a**, **b**) demonstrating radiotracer uptake at tumor site.** c** Sacral uptake indicating metastasis

Post embolization, the patient began experiencing intermittent left-sided chest/abdominal pain, mild fatigue, and a decrease in appetite. Six months after initial presentation, the patient was referred for a second opinion for further management of her tumor. At that time, a Ga-68 dotatate PET scan revealed tracer uptake in the sacrum, demonstrating osseous metastasis (Fig. 2).

Because of the progression and metastasis in the sacrum, it was recommended that the patient be considered for the clinical trial of lutetium Lu 177 dotatate (Lutathera) or be started on combination chemotherapy with cyclophosphamide, vincristine, and dacarbazine (CVD). The patient was deemed ineligible by the clinical trial institution and was thus started on systemic therapy with octreotide long-acting release (LAR) and oral temozolomide per institute recommendation. Clinical restaging done following gallium scan was stage IV (cT4, cNX, cM1c). Chemotherapy treatment was begun 9 months after initial presentation. Around this time, the patient began experiencing increased pain of the lower back/abdomen and intense fatigue that affected her daily activities. Palliative radiation therapy of the sacrum was initiated to manage metastasis and alleviate pain. Two months after beginning systemic therapy, the patient was admitted to the emergency room for acute altered mental status, diarrhea, anorexia, and ascites caused by suspected acute liver failure. As temozolomide was considered a differential along with passive congestion of the liver, the medication was discontinued to prevent further liver toxicity.

**Fig. 3:**
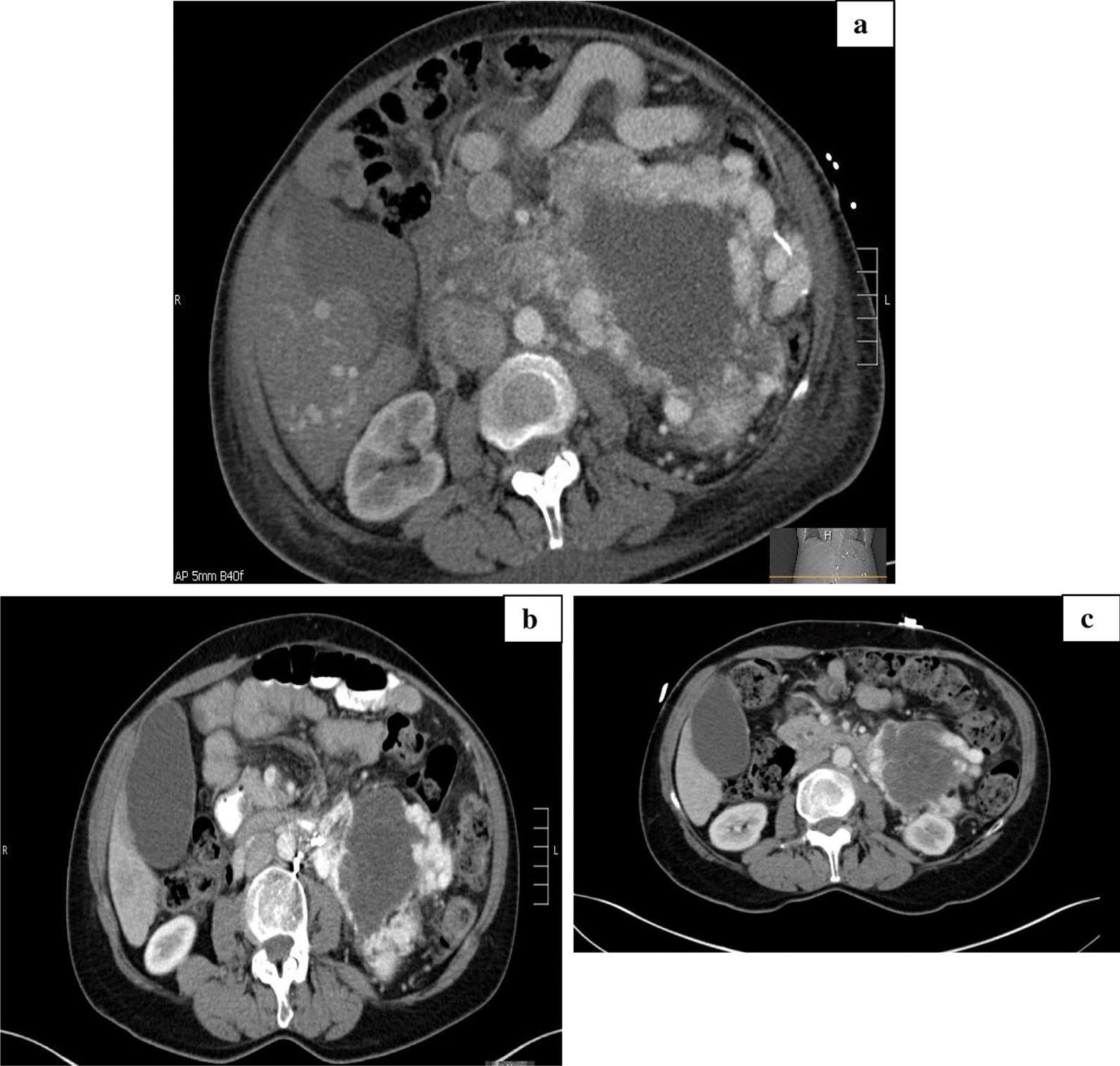
**a** Computed tomography scan pre-immunotherapy treatment, **b** CT restage IO × 5 months, and **c** CT restage IO × 8 months

Following stabilization, the patient was considered for the dual anti-cytotoxic T-lymphocyte-associated protein 4 (anti-CTLA-4) and anti-programmed death-ligand 1 (anti-PD-L1) in rare tumors (DART) clinical trial, which was studying the use of nivolumab and ipilimumab on rare tumors. Unfortunately, the PHEO/PGL arm was closed and thus patient was unable to take part. After discussions amongst multidisciplinary teams, the next best course of action was determined to be the use of off-label immunotherapy. Subsequently, 11 months after initial presentation, she was started on ipilimumab and nivolumab for salvage therapy in addition to octreotide LAR. Ipilimumab was administered at a dose of 1 mg/kg and nivolumab at a dose of 3 mg/kg. After four cycles of nivolumab and ipilimumab, the patient was tolerating the treatment well, with only the formation of a small pruritic rash. In addition, the patient began noticing decreased back pain and an increase in energy. A repeat PET revealed decreased size in tumor mass, reduced from 18.1 to 15.8 cm. A CT scan that followed corroborated the findings of decreased tumor size. On CT, the superior hypovascular portion of the mass was found to measure 7.4 × 5.7 cm compared with the initial 10 × 6.6 cm. The inferior hypervascular component also demonstrated a decrease in size to 6 × 6 cm from 7.8 × 7.6 cm. Subsequent CT scans conducted during immunotherapy treatment continued to illustrate decreased tumor mass size (Table 1 and Fig. 3).

**Table 1:** Mass size

Preimmunotherapy	Superior hypovascular Inferior hypervascular	10 × 6.6 cm 7.8 × 7.6 cm
Immunotherapy × 5 months	Superior hypovascular Inferior hypervascular	7.4 × 5.7 cm 6 × 6 cm
Immunotherapy × 7 months	Superior hypovascular Inferior hypervascular	7.5 × 5.2 cm 5.3 × 5 cm
Immunotherapy × 11 months	Superior hypovascular Inferior hypervascular	7.1 × 5.4 cm 4.8 × 4 cm
Immunotherapy × 13 months	Superior hypovascular Inferior hypervascular	7.3 × 5.5 cm 3.6 × 5.1 cm
Immunotherapy × 18 months	Superior hypovascular Inferior hypervascular	7.1 × 5.2 cm 3.8 × 3.7 cm

A repeat bone scan performed 1 year after initiating immunotherapy also showed no evidence of intraosseous metastasis of the sacral region. It was considered that the radiation therapy to the sacrum potentially contributed to the lack of metastasis on imaging studies. The patient remained stable on immunotherapy for approximately 15 months, with no serious adverse events occurring in that time. Following, she did experience some increase in abdominal pain, which a CT scan revealed to be due to development of a subocclusive thrombus. This was treated with change to another novel oral anticoagulant. The CT scan done at that time did not demonstrate any change in the pheochromocytoma.

**Fig. 4:**
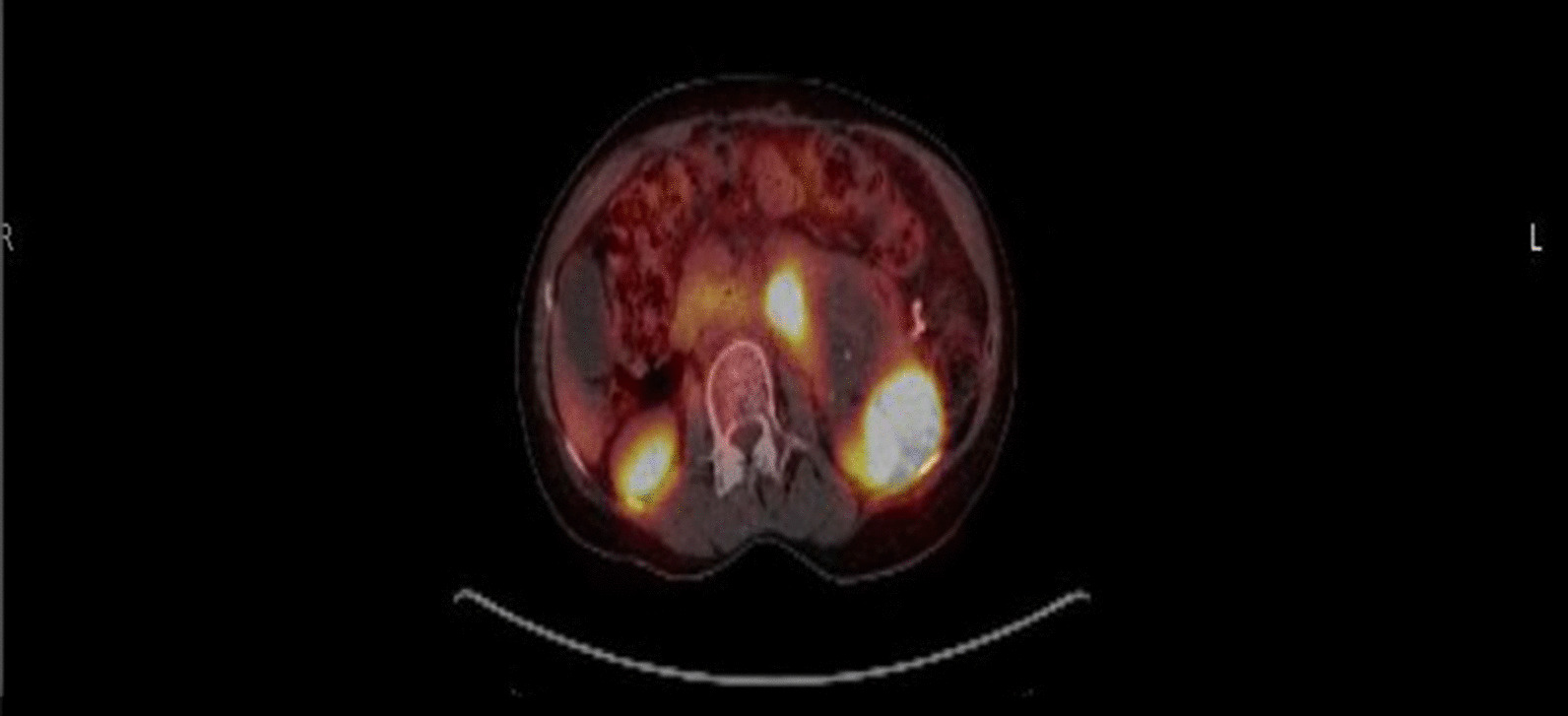
PET restaging scan demonstrating SSR positivity

As of today, the patient has been on immunotherapy maintenance treatment for over 20 months, and scans continue to demonstrate decreased size of the tumor mass. A repeat gallium-68 PET restaging study demonstrated somatostatin receptor (SSR) positivity of her tumor, which was useful for considering long-term management with octreotide (Fig. 4). She was also reevaluated and started on radiation therapy because of the considerable decrease in tumor size. She will continue to be on IO and octreotide during radiation therapy.

Overall, since initiation of immunotherapy treatment, the patient has exhibited a significant increase in quality of life, as she has resumed many of her normal activities and her abdominal/sacral pain has improved considerably. Also, she is now being afforded the opportunity for radiation therapy of the tumor mass. This is significant and promising as this was an option she was considered ineligible for prior to commencing IO treatment.

## Discussion

Pheochromocytomas and paragangliomas are rare neuroendocrine tumors [9]. The subset pheochromocytomas are those tumors found in the adrenal medulla, while the other paragangliomas are found within other neural crest cell-derived tissues of the body [9]. Because these tumors cause an increase in dopamine and norepinephrine release when functional, the symptoms include tachycardia, palpitations, flushing, abdominal pain, dyspnea, and nausea/vomiting. As these are a rare type of tumor, they make up only a small percentage of oncological cases each year. Out of these, malignant pheochromocytomas are even rarer, and there is a paucity of data regarding their management and outcomes [10]. Localized cases of these neuroendocrine tumors often revolve around controlling symptoms followed by surgical removal of the tumor.

Metastatic or locally unresectable cases of PHEOs/PGLs are often treated systemically with the use of combination chemotherapy and octreotide. In relation to the use of chemotherapy in PHEOs/PGLs, most research has been conducted on the use of combination chemotherapy with cyclophosphamide, vincristine, and dacarbazine (CVD) therapy [11]. The largest known retrospective study done on CVD treatment in the management of malignant PHEOs reportedly studied 52 patients and saw a 33% response rate to chemotherapy use with an additional 25% objective tumor response rate [11]. That study purported that the overall 5-year survival rate for patients with malignant PHEOs was 51% when using CVD therapy [11]. However, a recent meta-analysis reported that only approximately 37% of patients respond with the use of CVD therapy [5]. Out of the 37% of patients who had a response, there was a decrease in functional symptoms attributed to a decrease in tumor mass size as well as tumor stabilization [5].

As PHEOs/PGLs are neuroendocrine tumors, it has been well established that they often express multiple somatostatin receptors. Thus, octreotide is another management option for malignant PHEOs, especially those with somatostatin receptor positivity. Yet, studies conducted in regards to octreotide’s efficacy in PHEO management remain mixed. Some studies have demonstrated response in functional versions of the tumor, while other studies show no evidence of its benefit in controlling tumor functionality or causing tumor stability [11]. Despite the mixed response, various case reports can be found on use of octreotide in the management of PHEOs uncontrolled by other systemic therapies. This is inclusive of our case report detailed above [12]. Within our case report, octreotide along with IO maintained the patient for nearly 2 years. This is evidenced by a decrease in tumor size, which is noted in the table above, as well as the fact that imaging studies corroborate no evidence of current metastatic disease. The efficacy of octreotide in our patient may stem from her SSR positivity. As previously noted, octreotide plays a role in those tumors that express somatostatin receptors. In addition, our patient, who was intolerant of chemotherapy, has been doing well on both octreotide and IO. She is also now afforded the opportunity to have radiation therapy on the tumor itself because of the decrease in size. The patient also noted that, since beginning both IO and octreotide management, her quality of life has increased, as she is able to perform activities that she no longer could prior to these combined treatment options. Because of its effects on angiogenesis, along with various studies that still support its efficacy in malignant PHEO/PGL management, especially those with SSR positivity, octreotide still remains a viable option that should be considered for unresectable/metastatic cases.

Newer therapies that are being studied and used in management of PHEOs/PGLs include Lu-dotatate, also known as Lutathera. It has already been approved for SSR-positive gastropancreatic neuroendocrine tumor management [5]. This agent is part of the peptide-receptor radionuclide therapy (PRRT) agents and works by binding to the somatostatin receptors within the PHEOs/PGLs and emitting radiation directly into the receptors [5]. Like octreotide, the studies that have thus far been conducted for Lutathera efficacy in the use for malignant PHEOs/PGLs remain mixed. Clinical trials are still ongoing in testing the use of Lutathera in rare tumors, including PHEOs/PGLs [13]. Initially, the first reports did not show much promise for Lutathera, with only a 10% response rate for patients with malignant PHEOs/PGLs [5]. More recent studies, however, have illustrated greater benefit in this patient population [5]. According to Jimenez (2018), a recent retrospective study of 20 patients with malignant PHEOs/PGLs demonstrated a 29% partial response rate, with 62% of the patients demonstrating stability in their disease progression with 3 months of treatment [5]. Another study of 28 patients also demonstrated disease stability in 71% of the patients [11].

Another agent being studied for its effects in malignant PHEOs/PGLs is tyrosine kinase inhibitors (TKIs). The first TKI studied in malignant PHEO/PGL patients was sunitinib, followed shortly thereafter by pazopanib [5]. Both of these TKIs work in a similar manner by blocking vascular endothelial growth factor (VEGF), thereby preventing neoangiogenesis and cellular growth [5]. Some of the first reports on the efficacy of TKIs against malignant PHEOs/PGLs were published in 2008 in the case of a patient with malignant PHEO related to VHL [14]. The report details that, following initiation of the TKI, there was a decrease in tumor size as well as a lessening of functional tumor symptoms [14]. Shortly thereafter, a retrospective study of 17 patients with malignant PHEOs that were nonresponsive or had progression with chemotherapy were started on sunitinib [5]. Of the 17 patients, 10 were evaluated and shown to have an objective response rate of 30% while on the TKI [5]. In this retrospective study, it was also noted that four patients with bone metastases who were started on sunitinib also demonstrated decreased uptake on subsequent PET imaging studies [5]. Evidence continues to mount for the use of TKIs in malignant PHEOs/PGLs. More recent studies have demonstrated that the effect of TKI in management is due not only to its antiangiogenic property but also due to the direct effects on tumor cell growth [15]. Because of these promising findings, more phase II trials are underway examining the use of TKI for management of malignant PHEOs/PGLs [16].

In regard to IO, this is another treatment option that is gaining momentum in its use for malignant PHEOs/PGLs. Immunotherapy acts as a biological agent that works by suppressing, inhibiting, or activating the immune responses of the body [17]. For example, pembrolizumab and nivolumab, both of which are being studied for their use in malignant PHEOs/PGLs, work by acting on PD-L1 receptors. These receptors bind to the PD-L1 ligand on T cells, thereby blocking the PD-L1 pathway that normally acts by downregulating the immune response. A recent phase II trial was conducted with the use of pembrolizumab on advanced rare cancers that had tumor progression with standard therapies [8]. Within this study, malignant PHEOs/PGLs were evaluated and demonstrated a 43% nonprogression rate and a 75% clinical benefit rate among the patients who were administered pembrolizumab [8]. It is important to note that the patients in this phase II trial were considered because they were either unresponsive to or intolerant of CVD chemotherapy treatment of their malignant PHEOs/PGLs. Although there were only eight patients studied in the PHEO/PGL arm of this clinical trial, the findings were considered significant [8]. These patients, similar to our own in this case report, had very few other options and had shown no progress on other therapies [8]. It is interesting to note within this clinical trial that the researchers demonstrated a clinical benefit rate of 75% as well as a nonprogression rate of 43%, yet did not demonstrate an objective response rate in any of the patients [8]. This finding is interesting because, within our described case report, the patient obtained an objective response that was clinically significant because there was a decrease in tumor size and she had a notable progression free survival rate of over 20 months on IO. Because of the promising results from immunotherapy, another phase II trial is also being conducted on the use of both nivolumab and ipilimumab with rare tumors such as malignant pheochromocytomas and paragangliomas. This trial is currently ongoing.

Although our case report only demonstrates the particular efficacy of the use of immunotherapy in one patient, it stands to reason that, with no significant side effects of the medication along with demonstrable improvements not only in tumor burden but in quality of life, more research needs to be done in regard to immunotherapy use within this population of patients. The results of not only the aforementioned clinical trial involving the use of pembrolizumab but also within our case report itself lend to the validity of considering novel systemic therapies in patients that have otherwise shown no response to or who are intolerant of chemotherapy treatment. Even as immunotherapy agents are often used to target tumor agnostic cancers with specific molecular markers, the functionality of these agents allows them to be used in even those cases that may not demonstrate unique markers. As mentioned previously, the patient described in our case report demonstrated no known germline mutations or molecular markers of the paragangliomas yet still had appreciable response to immunotherapy, thereby allowing for consideration of these agents even in patients not exhibiting molecular alterations.

Overall, the patient in our case report has demonstrated a progression-free survival rate of over 20 months, improved quality of life, decreased tumor burden, and the opportunity to start radiation therapy as a result of the effects of IO and octreotide on her tumor. This corroborates other studies, demonstrating the importance of IO in malignant PHEO/PGL cases, and should serve to further research on treatment options of these rare oncological cases.

## Conclusion

This case report details the use of immunotherapy in a 60-year-old woman with pheochromocytoma ineligible for surgery or radiation and who was intolerant to chemotherapy. This case report demonstrates that immunotherapy use can result in a significant decrease in tumor burden, as repeat CT scans illustrated a decreased size in the tumor mass following IO treatment. This report also demonstrates that immunotherapy can provide long-term disease control, giving credence to the need for more research into the use of immunotherapy in malignant pheochromocytomas and paragangliomas.

## Data Availability

Not applicable.

## Abbreviations

IO: Immunotherapy
CT: Computed tomography
PHEO: Pheochromocytoma
PGL: Paraganglioma
MIBG: Meta-iodobenzylguanidine
T: Tumor
N: Lymph node
M: Metastasis
mg/kg: Milligrams per kilogram
